# Association Between Polygenic Risk Score and Risk of Myopia

**DOI:** 10.1001/jamaophthalmol.2019.4421

**Published:** 2019-10-31

**Authors:** Neema Ghorbani Mojarrad, Denis Plotnikov, Cathy Williams, Jeremy A. Guggenheim

**Affiliations:** 1 School of Optometry and Vision Sciences, Cardiff University, Cardiff, United Kingdom; 2 Population Health Sciences, Bristol Medical School, University of Bristol, Bristol, United Kingdom

## Abstract

**Importance:**

Myopia is a leading cause of untreatable visual impairment and is increasing in prevalence worldwide. Interventions for slowing childhood myopia progression have shown success in randomized clinical trials; hence, there is a need to identify which children would benefit most from treatment intervention.

**Objectives:**

To examine whether genetic information alone can identify children at risk of myopia development and whether including a child’s genetic predisposition to educational attainment is associated with improved genetic prediction of the risk of myopia.

**Design, Setting, and Participants:**

Meta-analysis of 3 genome-wide association studies (GWAS) including a total of 711 984 individuals. These were a published GWAS for educational attainment and 2 GWAS for refractive error in the UK Biobank, which is a multisite cohort study that recruited participants between January 2006 and October 2010. A polygenic risk score was applied in a population-based validation sample examined between September 1998 and September 2000 (Avon Longitudinal Study of Parents and Children [ALSPAC] mothers). Data analysis was performed from February 2018 to May 2019.

**Main Outcomes and Measures:**

The primary outcome was the area under the receiver operating characteristic curve (AUROC) in analyses for predicting myopia, using noncycloplegic autorefraction measurements for myopia severity levels of less than or equal to −0.75 diopter (D) (any), less than or equal to −3.00 D (moderate), or less than or equal to −5.00 D (high). The predictor variable was a polygenic risk score (PRS) derived from genome-wide association study data for refractive error (n = 95 619), age of onset of spectacle wear (n = 287 448), and educational attainment (n = 328 917).

**Results:**

A total of 383 067 adults aged 40 to 69 years from the UK Biobank were included in the new GWAS analyses. The PRS was evaluated in 1516 adults aged 24 to 51 years from the ALSPAC mothers cohort. The PRS had an AUROC of 0.67 (95% CI, 0.65-0.70) for myopia, 0.75 (95% CI, 0.70-0.79) for moderate myopia, and 0.73 (95% CI, 0.66-0.80) for high myopia. Inclusion in the PRS of information associated with genetic predisposition to educational attainment marginally improved the AUROC for myopia (AUROC, 0.674 vs 0.668; *P* = .02), but not those for moderate and high myopia. Individuals with a PRS in the top 10% were at 6.1-fold higher risk (95% CI, 3.4–10.9) of high myopia.

**Conclusions and Relevance:**

A personalized medicine approach may be feasible for detecting very young children at risk of myopia. However, accuracy must improve further to merit uptake in clinical practice; currently, cycloplegic autorefraction remains a better indicator of myopia risk (AUROC, 0.87).

## Key Points

**Question:**

Can genetic information be used to predict children at risk of myopia development?

**Findings:**

In this meta-analysis of 3 genome-wide association studies, a polygenic risk score derived from 711 984 participants was evaluated in an independent validation sample of 1516 participants. The area under the receiver operating characteristics curve for predicting myopia was 0.67 and for predicting high myopia was 0.73; individuals with polygenic risk scores in the top 10% appeared to be at a 6.0-fold higher risk of high myopia.

**Meaning:**

A personalized medicine approach appears to be feasible for detecting very young children aged 0 to 6 years at risk of myopia; however, beyond the age of 6 years, cycloplegic autorefraction seems to perform better.

## Teaser

This meta-analysis of 3 genome-wide association studies examines the use of genetic information to predict the risk of myopia development in children in the United Kingdom.

## Introduction

Myopia is a refractive error that typically develops during adolescence to cause poor distance vision. It is associated with an increased risk of sight-threatening diseases, such as glaucoma, maculopathy, and retinal detachment.^1^ Successful randomized clinical trials of interventions designed to slow childhood myopia progression suggest it would be beneficial to identify children at high risk of myopia development, who would benefit most from early treatment intervention.^2,3,4,5^ Currently, the best predictor of children at risk is a low hyperopic refractive error at an age before myopia typically manifests,^6^ suggesting that a screening regimen of cycloplegic autorefraction would be an effective approach. However, the necessity for cycloplegia in young children makes this strategy resource and time expensive. Furthermore, as the transition from moderate to low hyperopia may be part of the process of myopia development, cycloplegic autorefraction screening may detect children too late if the aim is to instigate a prophylactic intervention.

Estimating disease risk using genetic prediction, that is, via a polygenic risk score (PRS), has the advantage of allowing at-risk individuals to be identified at any age from birth onward. No eye drops or physical assessments are required, and the same test of a blood or saliva sample can be used to predict a wide range of conditions.^7^

Currently, the best-performing PRS for refractive error explains 7.8% of the interindividual variance in the trait.^8^ This PRS was derived from 7307 genetic variants identified in a genome-wide association study (GWAS) for mean spherical equivalent (MSE) refractive error and age of onset of spectacle wear (AOSW) in 160 420 participants.^8^ Combining participant data for a trait of interest with participant data for 1 or more genetically correlated traits has been shown to improve genetic prediction of the trait of interest.^9,10^ Herein, we tested the hypothesis that combining participant data from genetic studies of refractive error and genetic studies of educational attainment—a trait that is genetically correlated with refractive error—would be associated with improvements in the accuracy of a PRS in predicting individuals at risk of myopia.

## Methods

Of the various approaches for creating a PRS, the following 4-step method has been shown to be effective^7,9^ and was adopted for the present study. In step 1, 1 or more GWAS analyses are performed for the trait of interest. In step 2, which is optional, if more than 1 GWAS has been performed, the summary results from the separate GWAS samples are meta-analyzed to increase the effective sample size. In step 3, account is taken for the nonindependence of genetic markers in local regions, termed *linkage disequilibrium* (LD). In step 4, the PRS for an individual in the test population, such as a child whose risk of myopia is unknown, is calculated by summing the LD-adjusted regression coefficient for each GWAS marker by the number of copies of the risk allele the individual carries.

The study adhered to the Declaration of Helsinki and was conducted in compliance with laws in the United Kingdom.^11^ All participants provided written consent either via a hard copy or electronic consent form. For the UK Biobank, ethical approval was obtained from the National Health Service National Research Ethics Service.^12^ For ALSPAC, written informed consent for the use of data collected via questionnaires and clinics was obtained from participants following the recommendations of the ALSPAC Ethics and Law Committee at the time.

Multitrait analysis of GWAS (MTAG) meta-analysis^9^ has been proposed as a method superior to conventional (eg, inverse variance weighted) meta-analysis for combining information from disparate traits in step 2. Herein, we sought to capture information about genetic risk from the associated traits autorefraction-measured MSE and AOSW-inferred MSE. The genetic correlation of a pair of related traits has been proposed as a guide to their utility in an MTAG-derived PRS.^9^

To find the best-performing PRS generated using either a single trait or multiple traits, we assessed PRSs generated from the following 3 GWAS samples or traits: (1) a GWAS for autorefraction-measured MSE, (2) a GWAS for AOSW-inferred MSE, and (3) a GWAS for years spent in full-time education (EduYears) (see third paragraph of Statistical Analysis section). Additional PRSs were then calculated after using MTAG to combine GWAS summary statistics for multiple traits. The performance of each PRS was evaluated in an independent validation sample.

### Validation Sample

The validation sample comprised mothers whose children were enrolled in the Avon Longitudinal Study of Parents and Children (ALSPAC).^12,13^ Pregnant women resident in Avon, England, with expected dates of delivery April 1, 1991, to December 31, 1992, were invited to take part in the study. The initial number of pregnancies enrolled was 14 541, and 13 988 children were alive at age 1 year. ALSPAC parents who attended the research clinic with their child or children for autorefraction at the age of 7 years were invited to undergo noncycloplegic autorefraction themselves. There was a total of 1516 ALSPAC mothers with autorefraction data and genotype data available.^13^ The ALSPAC study website contains details of all of the data that are available through a fully searchable data dictionary and variable search tool.^14^

### Statistical Analysis 

Step 1 involved GWAS analyses and meta-analysis of summary data. A GWAS for autorefraction-measured refractive error was carried out in 95 619 UK Biobank participants of European ancestry aged 40 to 69 years who underwent noncycloplegic autorefraction. Details of the sample and the GWAS parameters have been reported previously.^8,15^

A GWAS for AOSW-inferred MSE was carried out in 287 448 UK Biobank participants of European ancestry aged 40 to 69 years who did not undergo autorefraction; that is, there was no overlap with the autorefraction-measured MSE GWAS sample. The GWAS parameters were the same as those used for the GWAS for autorefraction-measured MSE. The AOSW-inferred MSE phenotype was derived using information about the participants’ age, sex, and self-reported AOSW (questionnaire item, What age did you first start to wear glasses or contact lenses?) as described in eMethods 1 in the Supplement. In out-of-sample validation tests, the coefficient of determination between autorefraction-measured MSE and AOSW-inferred MSE was *R*^2^ = 0.30. All statistical tests were 2-sided. We interpreted *P* values in accordance with the 2016 Position Statement of the American Statistical Association.^16^

Summary statistics for a GWAS for EduYears by Okbay et al^17^ in 328 917 participants were downloaded from the Social Science Genetic Association Consortium website.^18^ In step 2, GWAS summary statistics were combined using MTAG,^9^ which relies on the assumption that effects sizes for all single-nucleotide polymorphisms share the same variance–covariance matrix to combine GWAS summary statistics from different traits to generate trait-specific regression coefficients.^9^

Step 3 was designed to account for LD between GWAS markers. LDpred^19^ uses a bayesian model to calculate posterior mean effect sizes for each GWAS marker, based on the local LD pattern in an ancestry-matched reference sample. We used the LDpred, version 1.0.6, infinitesimal model for approximately 1.1 million HapMap3 variants, with 2500 randomly selected UK Biobank participants of European ancestry as the LD reference panel. Constructing a PRS using a very large number of genetic markers (eg, 1.1 million) has been reported to provide better prediction performance than simply selecting the most strongly associated GWAS markers.^7,10,19^

A PRS was calculated for each of the 1516 ALSPAC mothers in the validation sample, using the regression coefficients generated by LDpred for 1.1 million HapMap3 variants. The mother’s refractive error (noncycloplegic autorefraction measure) was regressed on the standardized PRS to calculate the variance explained (*R*^2^) by the PRS (eMethods 2 in the Supplement). Similarly, the area under the curve (AUC) of the receiver operating characteristics (ROC) curve was calculated in the validation sample for 3 myopia severity levels: any myopia (autorefraction-measured MSE ≤ −0.75 diopter [D]), moderate myopia (autorefraction-measured MSE ≤ −3.00 D), and high myopia (autorefraction-measured MSE ≤ −5.00 D). To test for an improvement in the AUC of 2 ROC curves, we used the roc.test function from the R, version 3.5.0 package pROC (R Foundation) (eMethods 3 in the Supplement).^20^ We also calculated the odds ratio for myopia for participants in the top 25th, 10th, and 5th percentiles of the PRS vs the remaining participants (eMethods 4 in the Supplement). For comparison with the PRS derived from 1.1 million HapMap3 variants, we also assessed the accuracy of a PRS derived from just the most strongly associated GWAS variants. This assessment was carried out using *P* value–based clumping and thresholding (so called P+T) using the default settings of the PRSice software^21^ (modified to include the X chromosome).

The genetic correlation of each trait with autorefraction-measured MSE was determined with LDscore regression,^22^ using the GWAS summary statistics for each trait. Statistical analyses were performed using R, version 3.5.0 (R Foundation). All *R*^2^ values quoted are adjusted *R*^2^. Data analysis was performed from February 2018 to May 2019.

## Results

### Genetic Correlations

The genetic correlation between autorefraction-measured MSE vs the AOSW-inferred MSE phenotype was *r*_g_ = +0.92, consistent with the close association between an early AOSW and a more negative refractive error. The genetic correlation between autorefraction-measured MSE vs EduYears was *r*_g_ = −0.26, and for AOSW-inferred MSE vs EduYears, *r*_g_ = −0.35. The negative correlation resulted from higher educational attainment being associated with a more negative refractive error.^23^ The magnitude of these genetic correlations was deemed sufficient to warrant testing the hypothesis that integrating GWAS summary data for refractive error and EduYears would increase the accuracy of a PRS for refractive error and myopia.

### Accuracy of a PRS for Prediction of Refractive Error

**Figure 1.**
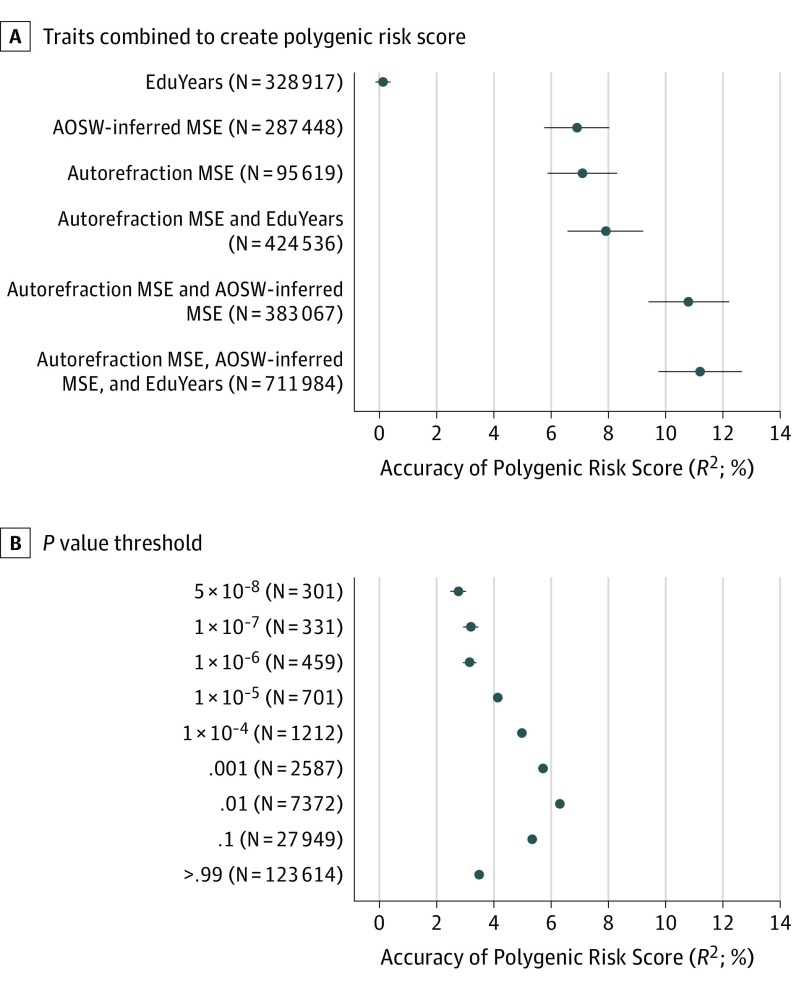
Accuracy of Predicting Refractive Error Using a Polygenic Risk Score (PRS) A, The PRSs were created using genome-wide association study (GWAS) summary statistics for the traits, autorefraction-measured mean spherical equivalent (MSE), age of onset of spectacle wear (AOSW)-inferred MSE, and years spent in full-time education (EduYears), either individually or combined by multi-trait analysis of GWAS (MTAG) meta-analysis. Each PRS included information from approximately 1.1 million genetic variants. The number in parentheses is the combined number of individuals in the GWAS analyses used to create the PRS. B, The PRSs were derived by *P* value–based clumping and thresholding (also known as P+T) using PRSice. Summary statistics from the MTAG meta-analysis of GWAS for autorefraction-measured MSE, AOSW-inferred MSE, and EduYears were used as input. The y-axis indicates the *P* value threshold used for the thresholding step. The number in parentheses is the number of genetic variants used to create the PRS. *R*^2^ values are the adjusted *R*^2^. Error bars represent the SE.

Accuracy in predicting refractive error was assessed in the all-female validation sample using a PRS derived from each GWAS trait alone (ie, autorefraction-measured MSE, AOSW-inferred MSE, or EduYears) or multiple GWAS traits combined using MTAG meta-analysis, as shown in Figure 1A and Table 1. In all of these analyses, the PRS for each participant was generated using genotypes for 1.1 million variants. The PRS derived from the EduYears GWAS alone provided limited predictive accuracy (*R^2^* = 0.14%), while the PRS derived from either autorefraction-measured MSE alone or AOSW-inferred MSE alone had a similar level of performance (*R^2^* = 7.1% vs *R^2^* = 6.9%). Including any 2 of the traits together improved performance, with the best result obtained for autorefraction-measured MSE combined with AOSW-inferred MSE (*R*^2^ = 10.8%). For all 3 traits combined, predictive performance improved further compared with the PRS without EduYears (*R*^2^ = 11.2% vs 10.8%; model fit *P* = .005). Adjustment for the participants’ age prior to estimating the accuracy of the PRS did not substantially change these *R*^2^ values.

**Table 1.** Polygenic Risk Scores for Predicting Refractive Error, Derived From Each of 3 GWAS Traits Separately or in Combination^a^

Trait	No. of Individuals^b^	MTAG Performed	Polygenic Risk Score *R*^2^, %^c^
Autorefraction-measured MSE	95 619	No	7.1
AOSW-inferred MSE	287 448	No	6.9
EduYears	328 917	No	0.1
Autorefraction-measured			
MSE and AOSW-inferred MSE	383 067	Yes	10.8
MSE and EduYears	424 536	Yes	7.9
MSE, AOSW-inferred MSE, and EduYears	711 984	Yes	11.2

Abbbreviations: AOSW, age of onset of spectacle wear; EduYears, years spent in full-time education; GWAS, genome-wide association studies; MSE, mean spherical equivalent; MTAG, multi-trait analysis of GWAS.

^a^ Traits included autorefraction-measured MSE, AOSW-inferred MSE, and EduYears.

^b^ The combined number of individuals in the GWAS analyses used to create the polygenic risk score.

^c^ All polygenic risk scores were created using genotype data for approximately 1.1M genetic variants. *R*^2^ values are the adjusted *R*^2^.

For comparison with the previously mentioned PRSs that incorporated 1.1 million genetic variants, we also evaluated the performance of simpler PRSs derived from just the most strongly associated GWAS variants. The highest accuracy (*R*^2^ = 6.3%) was obtained with a *P* value threshold of .01 for a PRS derived from 7372 variants (Figure 1B).

### Performance of a PRS for the Prediction of Myopia

**Figure 2.**
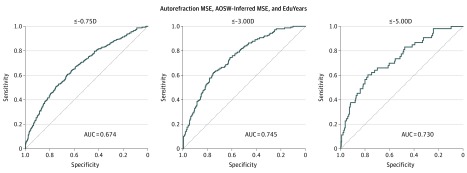
Receiver Operating Characteristic Curves for Detecting Myopia Using Polygenic Risk Scores Polygenic risk scores were calculated by combing information from genome-wide association study summary data for the traits, autorefraction-measured mean spherical equivalent (MSE), age of onset of spectacle wear (AOSW)-inferred MSE, and years spent in full-time education (EduYears). AUC indicates area under the curve.

**Figure 3.**
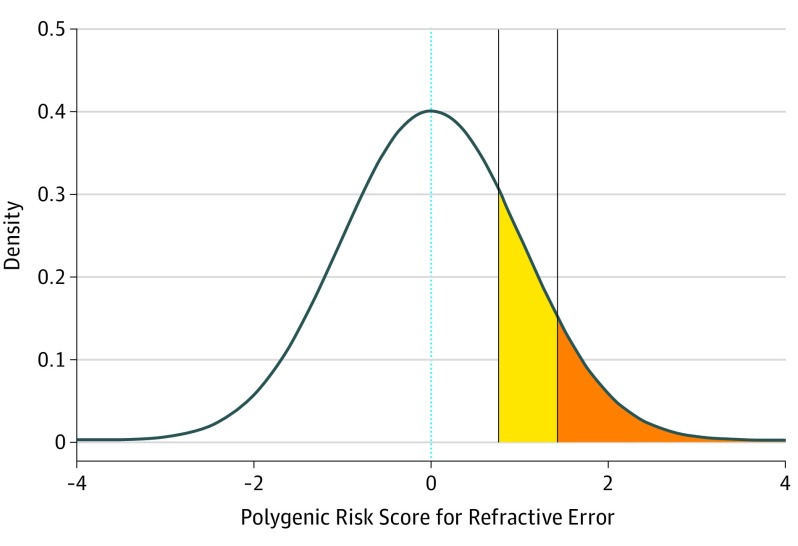
Distribution of the Polygenic Risk Score (PRS) The values of the PRS have been standardized to aid interpretation. The PRS was derived from the combined summary statistics of autorefraction-measured mean spherical equivalent, age of onset of spectacle wear–inferred mean spherical equivalent, and years spent in full-time education. The odds ratio for high myopia was more than 4 times for individuals in the top 25th percentile of the PRS (yellow and orange shaded regions combined) vs the remainder of the population. The odds ratio for high myopia was more than 6 times for individuals in the top 10th percentile of the PRS (orange shaded region) vs the remainder of the population. The dotted blue line indicates a standardized polygenic risk score of 0.

The ROC curves and their corresponding AUC for the prediction of low, moderate, and high myopia in the validation sample are displayed in Figure 2 and the eFigure in the Supplement. Consistent with the performance in predicting refractive error, the PRS derived from the MTAG-based meta-analysis of autorefraction-measured MSE, AOSW-inferred MSE, and EduYears GWAS data demonstrated the highest area under the receiver operating characteristic curve (AUROC). This 3-trait PRS had AUROC values of 0.67 (95% CI, 0.65-0.70) for any myopia, 0.75 (95% CI, 0.70-0.79) for moderate myopia, and 0.73 (95% CI, 0.66-0.80) for high myopia. There was weak statistical support that the inclusion of GWAS summary data for EduYears marginally improved the AUROC for myopia (PRS derived with vs without information from the GWAS: 0.674 vs 0.668; *P* = .02), but not the AUROC for moderate (0.745 vs 0.742; *P* = .61) or high (0.730 vs 0.730; *P* = .98) myopia. Individuals with risk scores in the upper percentiles of the 3-trait PRS had an increased risk of myopia. Specifically, those with a PRS in the top 25% were at 3.0-fold to 5.0-fold higher risk of low, moderate, and high myopia, those in the top 10% were at 3.5-fold to 6.0-fold higher risk, while those in the top 5% were at 4.5-fold to 6.5-fold higher risk (Table 2; Figure 3). Of these results, arguably the most striking was that individuals with a PRS in the top 10% were at a 6.1-fold higher risk (95% CI, 3.4–10.9) of high myopia compared with the remaining 90% of the sample. Adjusting for the participants’ age did not substantially change these AUC values.

**Table 2.** Odds Ratios for Myopia of at Least −0.75, −3.00, and −5.00 Diopters (D) for Individuals Classified as High Risk Using a Polygenic Risk Score

Myopia Level	Group, %		Odds Ratio (95% CI)	*P* Value
	Risk	Reference		
≤−0.75 D	Top 25	Remaining 75	3.06 (2.40-3.91)	1.75 × 10^−19^
	Top 10	Remaining 90	3.47 (2.43-4.91)	9.70 × 10^−13^
	Top 5	Remaining 95	4.57 (2.84-7.51)	7.11 × 10^−10^
≤−3.00 D	Top 25	Remaining 75	4.66 (3.06-7.03)	3.93 × 10^−13^
	Top 10	Remaining 90	4.89 (3.41-7.06)	8.14 × 10^−18^
	Top 5	Remaining 95	5.42 (3.17-9.03)	1.95 × 10^−10^
≤−5.00 D	Top 25	Remaining 75	4.90 (2.81-8.72)	3.22 × 10^−8^
	Top 10	Remaining 90	6.11 (3.36-10.87)	1.20 × 10^−9^
	Top 5	Remaining 95	6.50 (3.14-12.48)	1.37 × 10^−7^

Odds ratios were calculated by comparing those in the high-risk group with the remainder of the population.

## Discussion

The results of this study suggest that, through advances in genetics, it is now possible to categorize a sample of children into 3 groups: a group at low risk of myopia and high myopia development, making up 75% of the sample; a high-risk group, comprising 25% of the sample who are at 3.0-fold to 5.0-fold increased risk; and a very high-risk subsample of the latter group, compromising 10% of the sample who are at 5.0-fold to 6.0-fold increased risk. The latter 2 groups may benefit most from an intervention to reduce the incidence of myopia, such as time outdoors, or to reduce the rate of myopia progression, such as orthokeratologic or atropine treatment. Nevertheless, genetic prediction remains far from perfect (the best AUROC was 0.75 for predicting moderate myopia) and it was less accurate than the previously reported approach of screening for a low level of hyperopia by cycloplegic autorefraction.^6^ For instance, in the Collaborative Longitudinal Evaluation of Ethnicity and Refractive Error study, the criterion of a cycloplegic autorefraction less than or equal to +0.75 D at age 6 years had an AUROC value of 0.87 for predicting incident myopia.^6^ The key advantage of genetic prediction over cycloplegic refraction is that the genetic approach could be used at very young ages, before any reduction in childhood hyperopia begins. However, as no prophylactic treatment intervention is currently available for preventing incident myopia (other than the advice for children to spend more time outdoors), this advantage is currently likely to be of academic interest rather than sufficient to recommend any change in clinical practice.

From the perspective of generating the best PRS for predicting refractive error, our findings support the hypothesis that inclusion of GWAS summary data for educational attainment is beneficial. For example, the accuracy of a PRS was improved (*R*^2^ = 10.8% vs 11.2%, model fit *P* = .005), as was the AUROC for predicting myopia (AUROC = 0.674 vs 0.668, *P* = .02). Nevertheless, these gains were modest considering the large sample size of the EduYears GWAS (n = 328 917). Consistent with earlier work,^9,10,19^ we found that a PRS derived from just the top GWAS variants was less accurate than the PRS derived from variants distributed across the whole genome (*R*^2^ = 6.3% for the PRS derived from 7372 top GWAS variants vs *R*^2^ = 11.2% for the PRS derived from 1.1 million variants) (Figure 1). In comparison with prior research to develop a PRS for refractive error, Tedja et al^8^ reported the previously best-performing PRS (*R*^2^ = 7.8% in adults), which was derived from 7307 genetic variants. Ghorbani Mojarrad et al^24^ reported that a PRS derived using the top 149 GWAS variants identified by Tedja et al^8^ had *R*^2^ values of 1.1% and 2.6% in children aged 7 and 15 years, respectively. By contrast, the same 149-variant PRSs had an *R*^2^ of 4.0% in the adult validation sample that we studied herein. Thus, predicting refractive error in childhood appears to be a more difficult task than predicting the refractive error an individual will attain by adulthood. However, the lack of cycloplegia in the Ghorbani Mojarrad et al^24^ study sample would have contributed to the imprecision of the PRS in children.

### Limitations and Strengths

Our study has several limitations. First, it illustrates that, as expected from quantitative genetics theory, there are diminishing returns in the predictive accuracy of a PRS as the effective GWAS sample size increases. As mentioned in the preceding paragraph, at the GWAS sample sizes currently available, genetic prediction is unable to match the performance of cycloplegic autorefraction in predicting children at risk of myopia (AUROC = 0.67 vs 0.87).^6^ Another limitation was that we only studied participants of European ancestry. A PRS derived for use in one ancestry group is expected to perform poorly in a separate ancestry group.^25^ GWAS studies that recruit more participants of non-European ancestry will be beneficial to investigate prediction of refractive error and myopia in non-European ancestry populations. Strengths of the study were the use of very large GWAS samples for deriving the PRS, evaluation of the PRS in a completely independent validation sample, and measurement of refractive error using noncycloplegic autorefraction, which is the criterion standard approach for this age range (24-69 years).^26^

## Conclusions

Prediction of children at high (3.0-fold to 5.0-fold) and very high (4.5-fold to 6.5-fold) risk of myopia and high myopia was estimated using genetic information alone. Sensitivity and specificity did not reach the levels obtainable using cycloplegic autorefraction (AUC = 0.67 vs 0.87); however, genetic prediction offers the advantages of not requiring the use of eye drops and specialist clinical assessment and could be used to detect children who would benefit from interventions to prevent incident myopia (eg, more time outdoors) as well as to slow myopia progression. Incorporating genetic information capturing aspects of future educational attainment was found to be associated with improved accuracy of the PRS in predicting myopia, although the gain was modest. This work suggests that a personalized medicine approach to detecting children at risk of myopia is now feasible, although currently the accuracy of PRSs is not yet good enough to warrant their use in clinical practice.

## Notes

Supplement. eMethods 1. Statistical Model for Calculating AOSW-Inferred MSE eMethods 2. Assessing Accuracy of Refractive Error Prediction (R2) eMethods 3. Comparing the Fit of Two ROC Curves eMethods 4. Calculating the Odds Ratio for the Genetic Risk Score eFigure. ROC Curves for Detecting Myopia (≤−0.75 D,≤−3.00 D or≤−5.00 D) Using Polygenic Risk Scores
