# Supplementary material for: Association Between Polygenic Risk Score and Risk of Myopia
Source: JAMA Ophthalmol. 2019 Oct 31;138(1):7–13. doi: 10.1001/jamaophthalmol.2019.4421 (PMC6824229; doi:10.1001/jamaophthalmol.2019.4421)
Supplement: Supplement. — eMethods 1. Statistical Model for Calculating AOSW-Inferred MSE eMethods 2. Assessing Accuracy of Refractive Error Prediction (R2) eMethods 3. Comparing the Fit of Two ROC Curves eMethods 4. Calculating the Odds Ratio for the Genetic Risk Score eFigure. ROC Curves for Detecting Myopia (≤−0.75 D,≤−3.00 D or≤−5.00 D) Using Polygenic Risk Scores [file jamaophthalmol-138-7-s001.pdf]

Supplementary Online Content

Ghorbani Mojarrad N, Plotnikov D, Williams C,. Guggenheim JA; UK Biobank Eye and Vision Consortium. Association between polygenic risk score and risk of myopia. *JAMA Ophthalmol*. Published online October 31, 2019. doi:10.1001/jamaophthalmol.2019.4421

eMethods 1. Statistical Model for Calculating AOSW-Inferred MSE

eMethods 2. Assessing Accuracy of Refractive Error Prediction (R^2)

eMethods 3. Comparing the Fit of Two ROC Curves

eMethods 4. Calculating the Odds Ratio for the Genetic Risk Score

eFigure. ROC Curves for Detecting Myopia (≤ -0.75 D, ≤ -3.00 D Or ≤ -5.00 D) Using Polygenic Risk Scores

This supplementary material has been provided by the authors to give readers additional information about their work.

© 2019 Ghorbani Mojarrad et al. *JAMA Ophthalmology.*

eMethods 1. Statistical model for calculating AOSW-inferred MSE

The mean spherical equivalent (MSE) refractive error of participants who reported their age-at-onset of spectacle wear (AOSW) but who did not undergo autorefraction was calculated as follows. First, a statistical model was derived for participants who *did* undergo autorefraction and who reported their AOSW. Then, this model was used to predict the "AOSW-inferred MSE" of individuals who did not undergo autorefraction.

The statistical model took the form:

$$
\begin{aligned}
MSE_i = \mu + (\alpha \times Sex_i) + &\\
&\left(\beta_1 \times Age_i\right) + \left(\beta_2 \times Age_i^2\right) + \ \ldots. \ + \left(\beta_j \times Age_i^j\right) + \\
&(\delta_1 \times AOSW_i) + (\delta_2 \times AOSW_i^2) + \ \ldots. \ + \left(\delta_k \times AOSW_i^k\right)
\end{aligned}
$$

Where,

MSE_i is the autorefraction-measured refractive error of individual *i*

Sex_i is a binary variable indicating the gender of individual *i*

Age_i is the age in years of individual *i* when autorefraction was performed

$AOSW_i$ is the age-at-onset of spectacle wear in years of individual *i*

β_j are regression coefficients for polynomial terms *j* = 1, 2, 3, ..., *J*

β_k are regression coefficients for polynomial terms *k* = 1, 2, 3, ..., *K*

To optimize the model fit, the sample of participants who did undergo autorefraction and who reported their AOSW was split into a training sample and a test sample. Polynomials (*j*, *k*) of increasing order were fit using the training sample until the model fit showed no improvement. Specifically, nested models with polynomial order *j* vs. *j* + 1 (or *k* vs. *k* + 1) were compared using a likelihood ratio test (*LRT*), and polynomial order was increased until the LRT test suggested no improvement (*LRT* test, $p > 0$ .05). The performance of the final model was assessed by calculating the adjusted *R*-squared (squared correlation) between autorefraction-measured MSE and AOSW-inferred MSE in the test sample. The optimized model yielded an $R^2 = 0.30$.

© 2019 Ghorbani Mojarrad et al. *JAMA Ophthalmology.*

The inclusion of interaction terms did not improve model fit, nor did models using year-of-birth in place of *Age*. Models fit using a range of machine learning algorithms provided similar levels of performance. Models fit in samples with a truncated upper *Age* or *AOSW* ranges also produced similar performance, suggesting the absence of "tail wagging the dog" effects in the polynomial model.

© 2019 Ghorbani Mojarrad et al. *JAMA Ophthalmology.*

eMethods 2. Assessing accuracy of refractive error prediction (R^2)

Polygenic risk scores (PRS) were calculated for each of the 1,516 individuals in the Validation sample, using the formula:

$$PRS_i = \sum_{j=1}^{k} g_{ij}\beta_j$$

Where,

PRS_i is the PRS for individual *i* in the Validation sample

g_{ij} is the genotype of individual *i* for variant *j* (0, 1 or 2 copies of the minor allele)

β_j is the LDpred-adjusted regression coefficient for the minor allele of variant *j*

k is the total number of variants included in the PRS

Except for PRS calculated using threshold-based clumping, $k \approx 1.1$ million (the HapMap3 variants in the LDpred analyses). PRS were calculated for the following six GWAS trait combinations: (1) *Autorefraction-measured MSE*; (2) *AOSW-inferred MSE*; (3) *EduYears*; (4) *Autorefraction-measured MSE & AOSW-inferred MSE*; (5) *Autorefraction-measured MSE & EduYears*; (6) *Autorefraction-measured MSE, AOSW-inferred MSE & EduYears*.

To evaluate the accuracy of the PRS, a linear regression model was fitted, with refractive error (non-cycloplegic autorefraction measure) as the outcome (Y) and the PRS as the predictor (X). The adjusted R^2 (the variance in the true phenotype explained by the PRS) was taken as a measure of accuracy. The standard error of the R^2 was calculated using 2000 bootstrap replicates, using the R boot package.

To evaluate if the inclusion of GWAS summary statistics for *EduYears* in deriving the PRS improved the fit of a simpler model, e.g. to compare a PRS for *Autorefraction-measured MSE & EduYears* vs. *Autorefraction-measured MSE* alone, an ANOVA was performed for the following nested models:

© 2019 Ghorbani Mojarrad et al. *JAMA Ophthalmology.*

$$MSE_i = \mu + PRS_{Autorefraction\ MSE} \qquad Eq.1$$

$$MSE_i = \mu + PRS_{Autorefraction\ MSE} + PRS_{Diff} \qquad Eq.2$$

Where,

$$PRS_{Diff} = (PRS_{Autorefraction\ MSE\ \&\ EduYears} - PRS_{Autorefraction\ MSE})$$

This approach using nested models with vs. without the inclusion of PRS_{Diff} prevented multicollinearity between predictors (which could occur, for example, if both $PRS_{Autorefraction\ MSE\ \&\ EduYears}$ and $PRS_{Autorefraction\ MSE}$ were included in the same model). A p-value of $P < 0.05$ for the ANOVA comparison of $Eq.2$ vs. $Eq.1$ was considered evidence of an improvement in model fit.

© 2019 Ghorbani Mojarrad et al. *JAMA Ophthalmology.*

eMethods 3. Comparing the fit of two ROC curves

The roc.test function from the R package pROC was used to compare the fit of two ROC curves (for example, an ROC curve fit using a PRS derived from a GWAS for *autorefraction-measured MSE* vs. an ROC curve fit using a PRS derived from a GWAS for *autorefraction-measured MSE* and from a GWAS for *EduYears*).

Two thousand bootstrap replicates were drawn from the data, such that each replicate contained the same number of cases and controls as in the original sample. The difference in AUC of the two ROC curves was compared using *Eq.* 3 and the standard deviation of these difference was calculated using *Eq.* 4. Finally, a test statistic (D) was computed using *Eq.* 5 and compared against a standard normal distribution to obtain a p-value.

$$d_i = AUC1_i - AUC2_i \qquad Eq.3$$

$$s = \sqrt{\frac{\sum |\, d_i - \hat{d}\, |^2}{2000}} \qquad Eq.4$$

$$D = \frac{AUC1 - AUC2}{s} \qquad Eq.5$$

Where,

d_i is the difference in AUC in bootstrap replicate *i* = 1 ,2, ..., 2000

$AUC1_i$ is the AUC of the first model in bootstrap replicate *i*

$AUC2_i$ is the AUC of the second model in bootstrap replicate *i*

$\hat{d}$ is the mean difference in vector d_i for all 2000 bootstrap replicates

s is the standard deviation of vector d_i for all 2000 bootstrap replicates

$AUC1$ is the observed AUC for the first model

$AUC2$ is the observed AUC for the second model

D is the test statistic

© 2019 Ghorbani Mojarrad et al. *JAMA Ophthalmology.*

eMethods 4. Calculating the odds ratio for the genetic risk score

Odds ratios (OR) were calculated using logistic regression, according to the equation:

$$log \frac{p(Y = case|X)}{1 - p(Y = case|X)} = \beta_0 + (\beta_1 \times X)$$

Where,

$p(Y = case|X)$ is the probability of an individual being a classified as a case given their genetic risk X

β_0 is a regression coefficient quantifying the baseline risk when X = 0.

β_1 is a regression coefficient quantifying the risk due to X

X is a binary variable indicating genetic risk, equal to 1 if PRS > a threshold (e.g. top 5%) and equal to zero otherwise

Participants in the Validation sample were assigned as a case if their *autorefraction-measured MSE* was below a specific threshold: any myopia (*autorefraction-measured MSE* ≤ -0.75 D); moderate myopia (*autorefraction-measured MSE* ≤ -3.00 D); high myopia (*autorefraction-measured MSE* ≤ -5.00 D). All participants in the Validation sample were female, hence no term for gender was included in the logistic regression model. Inclusion of a term for age, corresponding to the age in years at which autorefraction was undertaken, did not appreciably alter the odds ratio for the PRS and hence this term was dropped from the model.

© 2019 Ghorbani Mojarrad et al. *JAMA Ophthalmology.*

eFigure 1. ROC curves for detecting myopia (≤ -0.75 D, ≤ -3.00 D or ≤ -5.00 D) using polygenic risk scores. Polygenic risk scores were calculated by combing information from GWAS summary data for the traits, *autorefraction-measured MSE*, *AOSW-inferred MSE,* and *EduYears*, either individually or combined.

See overleaf

© 2019 Ghorbani Mojarrad et al. *JAMA Ophthalmology.*

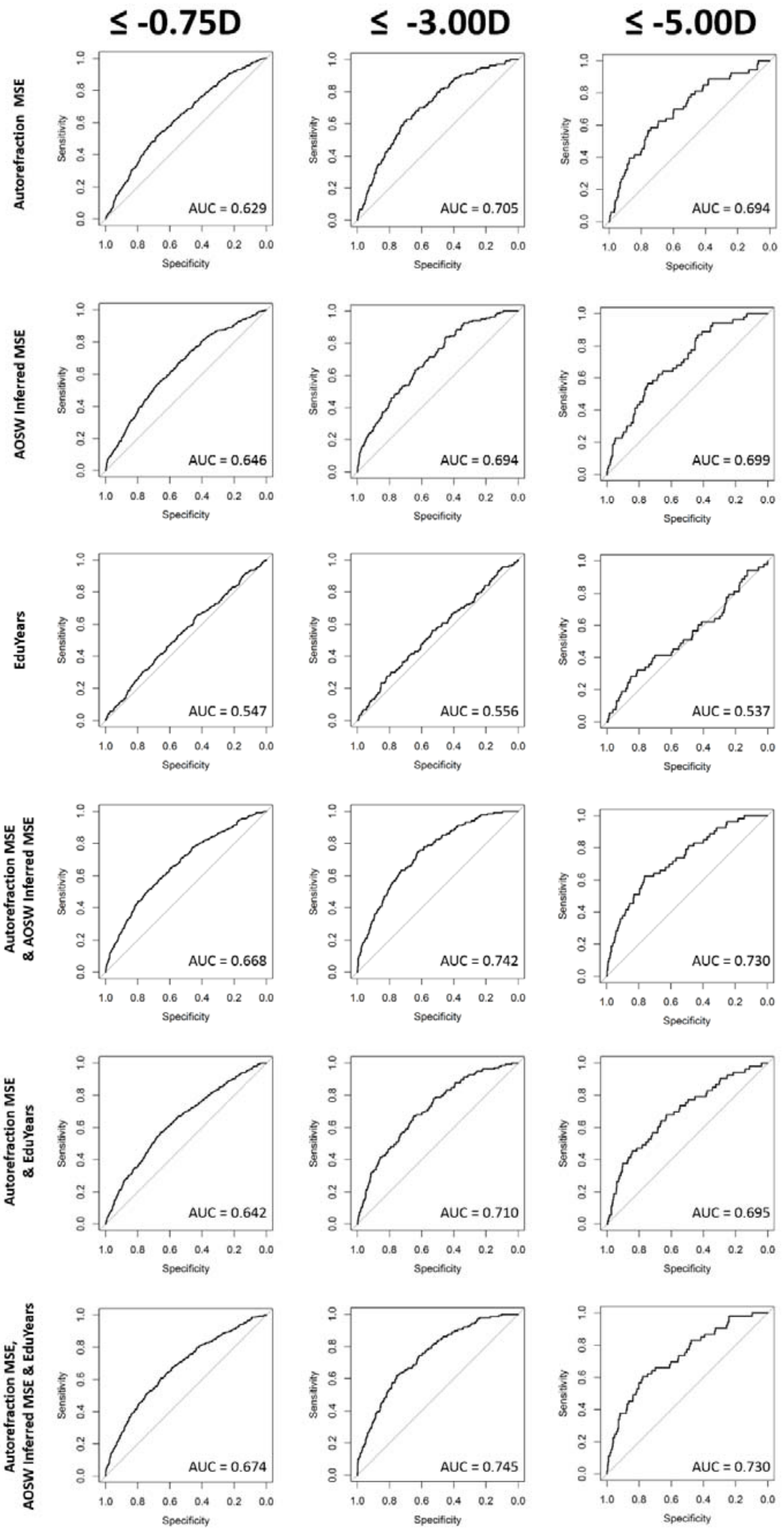

© 2019 Ghorbani Mojarrad et al. *JAMA Ophthalmology.*
